# Association between the Kihon Checklist and Working Status among Young-Old Citizens: A Cross-Sectional Study

**DOI:** 10.3390/geriatrics9040105

**Published:** 2024-08-20

**Authors:** Hironori Ohsugi, Saori Anzai, Yoshitaka Shiba

**Affiliations:** 1 Department of Physical Therapy, Faculty of Social Work Studies, Josai International University, Chiba 283-8555, Japan; 2 Department of Physical Therapy, School of Health Sciences, Fukushima Medical University, Fukushima 960-8031, Japan

**Keywords:** Kihon Checklist, working status, younger-older adults, daily functions

## Abstract

Owing to increasing super-aging societies, older adults will be encouraged to continue working. Although demand exists for older adults to continue working in Japanese society, the enabling factors have not been clarified. This study aimed to clarify (1) the life functions that affect the working status among younger-older adults who continue to work and those who do not via the Kihon Checklist (KCL) and (2) examine whether the number of areas of difficulties in daily functions of the KCL affected older adults’ employment status. This cross-sectional study involved 5386 older men and women aged 65 years or older in one city in Japan. Employment status and the seven domains of the Kihon Checklist (KCL) were analyzed. The KCL items related to employment status were the physical (odds ratio = 2.46, *p* < 0.01), socialization (odds ratio = 1.95, *p* < 0.01), and mood domains (odds ratio = 1.29, *p* < 0.01). Furthermore, the odds ratio increased to 2.06 when three or more domains were applicable. To remain employed, one must be physically and mentally healthy. Furthermore, since the risk of non-employment increased when one KCL domain was applicable, a broader assessment of life functions is necessary.

## 1. Introduction

The proportion of older adults is increasing worldwide. In particular, Japan is known as a super-aging country, with the aging population rate reaching 29% [1]. Owing to increasing super-aging societies, older adults will be encouraged to continue working. In 2021, the Japanese government enacted a law that keeps individuals under employment until they reach the age of 70 years. Continued employment of older adults will hopefully compensate for the shortage in the working population. A benefit for older adults themselves is the economic advantage of having an income.

Continuing to work in old age could reportedly maintain an individual’s health and reduce the risk of mortality [2]. Moreover, working in late life could provide long-term health benefits, such as reduced risk of poor self-rated health and limitations in activities of daily living (ADLs) [3], as well as maintain physical activity and mental function [4]. Thus, in addition to the social situation that enables older adults to continue working, there is also a demand for them to continue working from a health promotion perspective.

A previous study [5] argued that determinants of retirement planning in older workers are more important for their health than economic factors. However, some researchers suggest that economic stability plays a critical role in the decision to continue working. In Japan, the Kihon Checklist (KCL) [6] and Survey of Needs in Spheres of Daily Life (Survey of Needs) [7,8] have been widely used to evaluate various aspects of older adults’ health status. Both questionnaires, established by the Japanese Ministry of Health, Labour and Welfare (MHLW), assess older adults’ activities of daily living (ADLs). Furthermore, they can be used to assess physical functions, swallowing, mental functions, and various other aspects. The KCL has sufficient diagnostic accuracy for frailty and is considered useful for detecting older individuals at high risk [9]. It is also used to screen for long-term care insurance certifications among many occupations.

Although the demand for older adults to continue working exists in Japanese society, the enabling factors vary. Boot et al. [10] indicated that lower age, more weekly working hours, no functional limitations, fewer depressive symptoms, lower neuroticism scores, and higher sense of mastery were significantly associated with work. Therefore, since various factors are associated with continuation of work, a multifaceted assessment is required. The KCL is considered suitable as it allows for an assessment of various aspects related to ADLs. However, to our best knowledge, no previous studies have evaluated the KCL as a tool to assess continuation of work. Therefore, we clarified the life functions that affected employment status among young-older adults who continued to work and those of the same age who were not employed from a KCL perspective. This could identify the functions required for older adults to continue working. Furthermore, these findings could allow the KCL to be used as a tool to determine whether or not an older person can continue to work. If the KCL, a widely known and used tool in Japan, can be used to identify whether or not a person can work at an early stage, further exploration of the necessary approaches to keep older adults employed, which is required in today’s society, will be required.

## 2. Materials and Methods

### 2.1. Participants

This study analyzed secondary data that were collected via an administrative survey conducted by City A. The participants of the administrative survey included people aged 65 years and older (28,092) who resided in City A, as of 30 March 2017, and certified as not requiring care.

The respondents of the survey who met the selection criteria were included as participants in the study. The survey questionnaire, which comprised the KCL items, was mailed, and 21,586 responses were received. Inclusion criteria were those aged 69 years or younger, certified as not requiring support of long-term care insurance, and those who had no missing responses to the questionnaire items. Finally, 5386 responses were analyzed.

### 2.2. Survey Questionnaire

This survey used an original questionnaire developed by City A based on the KCL and Needs Survey. From the questionnaire, we selected answers from 25 items identical to those in the KCL. All items in the KCL were Yes/No answers; however, some items in the Needs Survey were multiple choice. Some items had three options: “can and does”, “can but does not”, and “cannot”. Items were reclassified into two values, with “cannot” as No and others as Yes. Regarding fall history, “many times” and “once” were coded Yes and “no” was coded as No. For fear of falling, “very fearful” and “somewhat fearful” were coded as Yes and “not so fearful” and “not fearful” were coded as No. For frequency of going out, “rarely go out” was coded as No and “once a week”, “2 to 4 times”, and “5 or more times a week” were coded as Yes. For decrease in frequency of going out, “very much less” and “less” were coded as Yes and “not much less” and “not less” were coded as No. After the numerical values were replaced, the items were divided into seven domains, identical to those in the KCL, according to the KCL’s scoring criteria [11]: “instrumental activities of daily living (IADLs)”, “physical”, “nutrition”, “eating”, “socialization”, “memory”, and “mood”. 

Each domain was considered as requiring support (applicable) when the scores exceeded a certain cut-off value. The IADL and physical domains ranged from questions 1 to 5 and 6 to 10, respectively. These were rated as “requiring support” for 3 or more negative responses [12]. Nutrition was defined as “requiring support” for negative responses to both questions (11 and 12) [11]. Eating was defined as “requiring support” for two or more negative responses to questions 13–15 [11]. Socialization was defined as “requiring” support for an answer of “no” to question 16 [11]. Although question 17 was also included, it was only a reference question [13]. Memory was defined as “requiring support” for one or more negative answers to questions 18–20 [11]. Mood was defined as “requiring support” for two or more negative answers to questions 21–25 [11].

### 2.3. Working Status

Working status was determined based on the question, “Are you working and how do you feel about working?” “Working” category was defined as “employed and would like to continue working” and “employed yet refraining from working”. Conversely, the “not working” category included the responses “not employed but would like to work” and “not employed and not considering working”. 

### 2.4. Statistical Analysis

We compared working status and age via a t-test. Furthermore, working and sex and each KCL domain were compared via a chi-squared (x^2^) test. A binomial logistic regression analysis (forward stepwise selection method) was performed with working (0) and not working (1) as the dependent variables and applicable (1) and not applicable (0) for each KCL domain as the independent variables. Age and sex were used as covariates in the adjusted models. Additionally, the sum of the number of applicable items in each KCL domain was calculated to generate four categories: none, one, two, and three or more. Subsequently, these were used as independent variables in the logistic regression analysis, with working/not working as the dependent variables and age and sex as covariates.

SPSS Statistics version 24.0 (IBM Japan, Tokyo, Japan) was used for all statistical analyses. Significance was set at *p* < 0.05.

### 2.5. Ethical Considerations

This study conducted a secondary analysis of data from an administrative survey from City A. All participants were informed that the collected data would be used for research purposes during data collection. Additionally, the researchers only received completely anonymized data, which did not contain participants’ personal information. This study was approved by the relevant Ethics Review Committee.

## 3. Results

### 3.1. Participants’ Characteristics

Table 1 presents the participants’ characteristics. Their mean age was 67.1 ± 1.4 years, and 47.0% were male. Table 2 presents the results of the comparison of each item according to employment status. The working group was younger (*p* < 0.01) and had a higher percentage of males (*p* < 0.01) than the not-working group. Additionally, the working group showed higher “not applicable” results in all the KCL domains, which indicated better functionality.

### 3.2. KCL Items Related to Working Status

Binomial logistic regression analysis revealed that the physical (odds ratio = 2.46, *p* < 0.01), socialization (odds ratio = 1.95, *p* < 0.01), and mood (odds ratio = 1.29, *p* < 0.01) domains were significant factors related to work. Furthermore, they remained significant in all models adjusted for age and sex (physical: odds ratio = 2.29, *p* < 0.01; socialization: odds ratio = 2.28, *p* < 0.01; and mood: odds ratio = 1.33, *p* < 0.01), as shown in Table 3.

### 3.3. Relation between Working Status and the Number of KCL-Applicable Items

In the binomial logistic regression analysis with the number of KCL-applicable items as the independent variables, all categories were significant in the model adjusted for age and sex. The odds ratio increased as the number of applicable items increased, which indicated a cumulative effect of multiple functional impairments on the likelihood of not working (Table 4).

## 4. Discussion

This study examined the effects of the KCL domains on the working status among younger-older adults aged 65–69 years. Our findings indicated that the group that worked at the time of the survey had a significantly higher number of “not applicable” respondents for each KCL domain, which suggested better functionality. Specifically, three domains—physical, socialization, and mood—were identified as being related to working status. Increase in the number of applicable domains was associated with not working, which highlighted the cumulative impact of multiple functional impairments.

Menai et al. [14] demonstrated that continued employment helped maintain physical activity, while retirees exhibited increased sedentary behavior. Hence, decline in physical activity associated with retirement could cause a decline in physical functions. Moreover, poor physical health was a common expected reason for retiring [15]. Some people retired owing to declining physical functions. Hasselhorn et al. [16] indicated that both good and poor health could lead to early or late retirement; however, they emphasized that the work was adapted to participants’ health limitations in all the cases. This suggested that people who continue working may be able to maintain their physical functions through their employment, and in turn, having sufficient physical functions may enable them to continue working. Conversely, among unemployed older adults, their physical functions could have declined as a result of quitting their job or because they were forced to quit their job due to a decline in their physical functions.

Regarding socialization, our findings aligned with of those of previous studies, which indicated that working older adults were more likely to go out frequently, which reduced the risk of becoming homebound [17,18]. Work was a form of social participation and provided a purpose in life and enhanced social contacts [19]. Furthermore, Menai et al. [14] reported that retirees may become more sedentary, which may cause them to become more withdrawn and participate less in social activities. Hence, socialization may be maintained by being employed.

Mood also significantly influenced working status, with those in better mental health more likely to remain employed. Van der Noordt et al.’s [20] systematic review revealed that employment was beneficial to health, especially in reducing depression and bettering general mental health. This suggested that the mental health of older adults who continued to work could have been preserved in our study as well. Conversely, other studies reported that work caused stress in employees and worsened their mental health. Van der Molen et al.’s [21] review indicated that work-related psychosocial risk factors were associated with a higher risk of stress-related mental disorders. Moreover, retirement was associated with more depressive symptoms [22]; older adults with depressive symptoms were unable to work. Therefore, while some people were able to maintain their mental health through employment, others were unable to continue working owing to depressive symptoms, which resulted in depressive symptoms that determined whether or not people could work.

The odds of not working increased with the number of applicable KCL domains, which indicated that while physical, socialization, and mood domains were the key factors, other domains also played a role. This underscored the importance of a comprehensive assessment of older adults’ health via the KCL to identify those at risk of non-employment. Working was related to various factors, such as mental health [20], physical activity [2], and social networks [23]. The KCL was used in various domains in Japan, such as to examine the current status of the older population and identify high-risk individuals in need of care. Therefore, many opportunities for its implementation exist and older adults’ risks for early retirement could be detected at an early stage. The implementation of an effective approach to identify those at employability risks via the analysis of regularly conducted KCL could promote the much-needed increase in the number of older workers in the future.

### 4.1. Implications

Our study highlights the necessity of addressing multiple health domains to support continued employment among older adults. Both physical and mental functions are crucial, and even one applicable KCL domain can impact employment status. Hence, a multifaceted approach to health assessment and intervention is essential for promoting employment in this demographic. Our results also suggest that the KCL may be used as an assessment tool for early detection of whether older people are able to continue working.

### 4.2. Limitations

This study has several limitations. First, the cross-sectional design precluded conclusions on causality between working status and health. Future longitudinal studies should determine whether poor health leads to unemployment or vice versa. Second, we did not collect detailed medical information, which could influence the ability to work. Additionally, economic factors were not considered, although they likely impacted employment decisions. Future research should incorporate medical and economic data to provide a more comprehensive understanding of the factors that affected older adults’ employment.

## 5. Conclusions

We demonstrated that it is possible to analyze the risk of employability according to the relevant areas of the KCL. Furthermore, the risk of inability to work increased as the relevant areas of the KCL increased. We believe that this study provides a new perspective on the use of the KCL in Japan, which has previously been used to analyze the risk of requiring long-term care.

## Figures and Tables

**Table 1:** Participants’ characteristics.

	All Participants (*n* = 5386)
Age, mean (SD), y	67.1 (1.4)
Sex (Male/Female), *n*	2533/2853
Working/Not working, *n*	2312/3074
KCL applicable/not applicable, *n*	
IADL domain	22/5364
Physical domain	149/5237
Nutrition domain	40/5346
Eating domain	651/4735
Socialization domain	102/5284
Memory domain	1482/3904
Mood domain	698/4688

Abbreviations: SD, standard deviation; KCL, Kihon Checklist; IADLs, instrumental activities of daily living.

**Table 2:** Comparison of items according to employment status.

	Working *n* = 2312	Not Working *n* = 3074	*p*
Age, mean (SD), y	67.0 (1.4)	67.2 (1.4)	<0.001 ^a^
Sex (Male/Female), *n*	1320/992	1213/1861	<0.001 ^b^
KCL applicable/not applicable, *n*			
IADL domain	2/2310	20/3054	0.001 ^b^
Physical domain	31/2281	118/2956	<0.001 ^b^
Nutrition domain	11/2301	29/3045	0.048 ^b^
Eating domain	252/2060	399/2675	0.020 ^b^
Socialization domain	24/2288	78/2996	<0.001 ^b^
Memory domain	603/1706	879/2195	0.041 ^b^
Mood domain	248/2064	450/2624	<0.001 ^b^

Abbreviations: SD, standard deviation; KCL, Kihon Checklist; IADLs, instrumental activities of daily living. ^a^ Student’s *t*-test; ^b^ χ^2^ test.

**Table 3:** Binomial logistic regression analysis for the KCL domains on not working.

	Crude Model			Adjusted Model		
	OR	95% CI	*p*	OR	95% CI	*p*
Physical domain	2.46	1.63–3.70	<0.001	2.29	1.52–3.47	<0.001
Socialization domain	1.95	1.22–3.12	0.006	2.28	1.42–3.68	0.001
Mood domain	1.29	1.08–1.52	0.004	1.33	1.12–1.58	0.001

Abbreviations: OR, odds ratio; CI, confidence interval; KCL, Kihon Checklist. Adjusted: age and sex.

**Table 4:** Odds ratio with number of KCL-applicable items.

	Crude Model			Adjusted Model		
	OR	95% CI	*p*	OR	95% CI	*p*
All negative	reference					
One domain positive	1.12	0.99–1.27	0.085	1.14	1.00–1.30	0.039
Two domains positive	1.40	1.15–1.71	0.001	1.49	1.21–1.82	<0.001
Three domains positive	1.96	1.45–2.64	<0.001	2.06	1.52–2.80	<0.001

Abbreviations: OR, odds ratio; CI, confidence interval; KCL, Kihon Checklist. Adjusted: age and sex.

## Data Availability

The data that support the findings of this study are available from City A, Japan, but restrictions apply to the availability of these data, which were used under license for the current study, and so are not publicly available.
